# C-Reactive Protein: Friend or Foe? Phylogeny From Heavy Metals to Modified Lipoproteins and SARS-CoV-2

**DOI:** 10.3389/fcvm.2022.797116

**Published:** 2022-03-24

**Authors:** Michael Torzewski

**Affiliations:** Department of Laboratory Medicine and Hospital Hygiene, Robert Bosch-Hospital, Stuttgart, Germany

**Keywords:** C-reactive protein, phylogeny, acute phase response, host defense, complement system, autoantigen, enzymatically modified LDL, SARS-CoV-2

## Abstract

Animal C-reactive protein (CRP) has a widespread existence throughout phylogeny implying that these proteins have essential functions mandatory to be preserved. About 500 million years of evolution teach us that there is a continuous interplay between emerging antigens and components of innate immunity. The most archaic physiological roles of CRP seem to be detoxication of heavy metals and other chemicals followed or accompanied by an acute phase response and host defense against bacterial, viral as well as parasitic infection. On the other hand, unusual antigens have emerged questioning the black-and-white perception of CRP as being invariably beneficial. Such antigens came along either as autoantigens like excessive tissue-stranded modified lipoprotein due to misdirected food intake linking CRP with atherosclerosis with an as yet open net effect, or as foreign antigens like SARS-CoV-2 inducing an uncontrolled CRP-mediated autoimmune response. The latter two examples impressingly demonstrate that a component of ancient immunity like CRP should not be considered under identical “beneficial” auspices throughout phylogeny but might effect quite the reverse as well.

## Introduction

In 1930, Tillet and Francis described a protein precipitating pneumococcal C-polysaccharide (CPS) in the sera of patients with various inflammatory diseases (1). Later on, human being turned out to be not the only vertebrate harboring such proteins as CRP-like precipitins were also found in plaice (*Pleuronectes platessa*) (2) and other marine teleosts demonstrating that CRP is an evolutionary conserved protein, which was defined by any two of the following three characteristics: (1) cyclic oligomer comprising similar subunits with a molecular weight of 20–30 kDa, (2) binding to phosphocholine (PCh) in a Ca^2+^-dependent manner, and (3) immunological cross-reactivity with human CRP (3). Given this definition, CRP had a widespread existence throughout phylogeny implying that these proteins have essential functions mandatory to be preserved (4). After about 500 million years of evolution both structure and function of CRP have evolved in collaboration with the entire immune system presumably with a gradually loss of its constitutive functions going along with increasing specific ligand-recognition functions. However, since the latter led to effector functions and evolutionary structure-function relationships of CRP are largely unknown, it is mandatory to understand the phylogeny of CRP function and the reasons behind. The aim of this paper was to review the functional spectrum of CRP during evolution (Table 1) contributing a phylogenetic viewpoint to the discussion whether CRP is functional in all humans and whether it is beneficial or harmful. The respective structure- and ligand binding-changes throughout phylogeny have been excellently reviewed recently (3) and are not a matter of the present review. Likewise, related proteins with similar structural and functional properties and therefore termed “pentraxins” by Osmand et al. (47) like serum amyloid P component or pentraxin-2 (SAP, PTX2) and long pentraxin (PTX3) are not considered herein.

**Table 1:** Phylogeny of CRP function.

**Taxon**	**Species**	**Antigen/target/systemic host defense response**		**What is CRP doing?**	**Ref**.
Arthropods	Horseshoe crab (*Limulus polyphemus*)	Chemicals	Mercury (Hg)	Scavenger	( 5 )
Molluscs	Giant African land snail (*Achatina fulica*)		Lead (Pb)	Reversion of the toxic effects	( 6 )
Teleosts	Rohu carp (*Labeo rohita*)		Cadmium (Cd), mercury (Hg), phenol, hexachlorocyclohexane	Elevated serum levels	(7, 8)
	Major South Asian carp (*Catla catla*)		Cadmium (Cd), mercury (Hg), phenol, hexachlorocyclohexane	Elevated serum levels of glyco- sylated molecular variants	(9, 10)
	Channel catfish (*Ictalurus punctatus*)		Turpentine oil	Acute phase pattern	( 11 )
	Rainbow trout (*Oncorhynchus mykiss*)		Turpentine oil	Significant decreases in the expression	( 12 )
			Formalin, metriphonate or potassium permanganate	Up and down of serum levels	( 13 )
	Plaice (*Pleuronectes platessa*)	Acute phase response	Adrenal hormons	Elevated serum levels	( 2 )
	Rainbow trout (*Salmo gairdneri*)		Temperature shock	Elevated serum levels	( 14 )
	Tongue sole (*Cynoglossus semilaevis)*	Bacteria and viruses, fungi, protists and metazoan parasites	Gram-negative pathogens (*Edwardsiella tarda, Vibrio/Listonella anguillarum, Escherichia coli*) Gram-positive pathogens (*Streptococcus iniae*)	Increasing respiratory burst and phagocytic capacity of peripheral blood leukocytes	( 15 )
	Common carp (*Cyprinus carpio*)		*Aeromonas spec. Escherichia coli*	Elevated serum levels	(16, 17)
	Black rockfish (*Sebastes schlegelii*)		Polysaccharides or live *Streptococcus iniae*	Significant upregulation in spleen and head kidney tissues	( 18 )
	Rainbow trout (*Salmo gairdneri*)		*Vibrio anguillarum* (intraperitoneal challenge)	Significant increase of the opsonising effect of CRP on macrophage phagocytosis	( 19 )
	Ayu (*Plecoglossus altivelis)*		*Vibrio anguillarum*	Significant upregulation, inhibition of complement 3 deposition on the bacteria further inhibiting comple- ment-mediated opsonophagocyto- sis by monocytes/macrophages	( 20 )
	Zebrafish (*Danio rerio*)		Spring viremia carp virus (SVCV)	Neutralization of viral infectivity	( 21 )
	Common carp (*Cyprinus carpio*)		Cyprinid herpesvirus 3 (CyHV-3)	Significant increase of CRP levels, distinct organ- and time-dependent expression profile patterns	( 22 )
	Goldfish (*Carassius auratus*)		*Trypanosoma carassii*	High expression in the kidney, liver and spleen at various days post infection, enhanced complement-mediated killing of trypanosomes *in vitro*	( 23 )
Mammals	Sprague Dawley rats	Chemicals	Raised level indicating acute tissue damage due to inflammation and necrosis caused by cadmium (Cd)		( 24 )
			sequestration and excretion of mercury (Hg)		( 25 )
	New Zealand white rabbits	Acute phase response	Typhoid vaccine	Accumulation at tissue sites of inflammation and necrosis	( 26 )
	Transgenic mice expressing rabbit CRP		Endotoxemia	Confers resistance	( 27 )
			Antigen-induced arthritis	onfers resistance	( 28 )
	Beagle dogs		Surgery	Increased and rapidly decreased with convalescence	( 29 )
	Human		Severe trauma	Production of anti-inflammatory cytokines by CD14(high)CD16(+) monocytes	( 30 )
	Rat, human	Bacteria and viruses, fungi, protists and metazoan parasites	*Plasmodium yoelii*	Inhibits *in vitro* development in hepatocytes	( 31 )
	Mouse		*Staphylococcus aureus*	Opsonine, involved in nonspecific resistance	( 32 )
	CRP transgenic mice		*Streptococcus pneumoniae*	Increased expression, protection by both phosphorylcholine (PCh)-dependent and PCh-independent mechanisms	(33–38)
	CRP deficient mice		*Streptococcus pneumoniae*	protection by reconstitution with isolated pure human CRP, no role of the classical complement pathway	(39, 40)
	Rat		*Schistosoma mansoni*	Platelets treated with CRP were capable of conferring significant protection against schistosomiasis in transfer experiments	( 41 )
	Human		SARS-CoV-2	Induces an uncontrolled auto- immune response and complement- and macrophage activation	(42, 43)
	Transgenic mouse and rabbit models	Neo-/autoantigens	Modified LDL	No effect, pro-atherogenic, anti-atherogenic	( 44 )
	Human		Modified LDL	Pro- or anti-atherogenic?	( 45 )
			Apoptotic cells	Promotes noninflammatory clear- ance of apoptotic cells	( 46 )

*Taxon: systematic animal group; Species: representative example of the taxon; Antigen: abiotic or biotic (differential colored) triggers of innate (or adaptive) immunity; What is CRP doing?: alterations of expression levels and/or assumed function*.

## Chemicals

One of the most archaic physiological roles of CRP seemed to be the detoxication of heavy metals and other chemicals. During phylogeny, it has been demonstrated already in arthropods and molluscs. Specifically, the CRP from horseshoe crab (*Limulus polyphemus*) bound mercury (Hg) both *in vivo* and *in vitro* maybe scavenging this heavy metal (5). Crossing the taxonomical barrier, giant African land snail (*Achatina fulica*) CRP both in total and individual subunits reversed the toxic effects (leading to oxidative stress and apoptosis) of lead (Pb) nitrate in rodents possibly due to scavenging of reactive oxygen species (6).

In terms of teleosts, cadmium (Cd), Hg, phenol, and hexachlorocyclohexane-polluted water led to three- to five-fold elevated levels of a pollutant specific molecular variant in the sera of Rohu carp (*Labeo rohita*) (7, 8). Likewise, elevated serum levels of pollutant specific Cd, Hg, phenol and hexachlorocyclohexane variants of CRP differing significantly in total carbohydrate contents were observed in South Asian carp (*Catla catla*) and other fishes exposed to the above mentioned chemicals (9, 10).

Channel catfish (*Ictalurus punctatus*) serum contained a protein similar to human CRP precipitating CPS dependent on calcium. This protein displayed an acute phase response after injection of the inflammatory agent turpentine oil (11). On the other hand, intracellular CRP synthesis in hepatocytes, head kidney macrophages and spleen lymphocytes of rainbow trouts (*Oncorhynchus mykiss*) significantly decreased after exposure to turpentine oil (12). Up and down of serum CRP levels were also observed in trouts exposed to anti-ectoparasitic chemicals formalin, metriphonate or potassium permanganate. The authors concluded that measurement of CRP levels in trout serum might be useful as a bioindicator of the state of health (13).

In white rats, a raised level of CRP indicated acute tissue damage due to inflammation and necrosis caused by Cd (24). In Hg-treated rats, CRP was found to be synthesized in the liver which, in turn, sequestered Hg resulting in the denaturation of the protein into subunits. The subunits retained the Hg and were released into the serum from where it got excreted (25).

## Acute Phase Response

Generalizing and translating this mechanism to the acute phase response, it was obvious that changing the plasma concentrations not only of certain divalent cations like iron but also proteins during inflammation was an essential component of both invertebrate and vertebrate immune responses (48). In plaice, even adrenal hormones increased CRP synthesis without an additional stimulus (2). In rainbow trout (*Salmo gairdneri*), a CRP-like macromolecule could also be rapidly induced both by chemical and physical stress (14).

In the rabbit, a “neo-ra(rabbit)CRP” cross-reactive with a free human CRP subunit (“neo-huCRP antigen”) accumulated at tissue sites of inflammation and necrosis 24 and 48 h after exposure to typhoid vaccine (26). Moreover, transgenic mice expressing rabbit CRP were resistant to endotoxemia (27) and development of antigen-induced arthritis (28). Finally, the concentration of CRP in normal beagle dogs after surgery showed a similar pattern as in human beings with acute increase and subsequent decrease during convalescence (29).

## Bacteria and Viruses, Fungi, Protists and Metazoan Parasites

Exposure of peripheral blood leukocytes (PBL) of tongue sole (*Cynoglossus semilaevis*) to Gram-negative and Gram-positive bacteria together with recombinant CRP led to a significant increase of respiratory burst and phagocytic capacity indicating that *Cynoglossus semilaevis* CRP is important for protection against bacterial infection (15). Significant elevation of CRP serum levels were also observed in carp (*Cyprinus carpio*) infected with *Aeromonas spec*. and/or *Escherichia coli* (16, 17). Black rockfishes (*Sebastes schlegelii*) exposed to polysaccharides or live *Streptococcus iniae* showed a significantly raised basal expression of CRP in both spleen and head kidney (18). Nevertheless, the detailed functions of CRP in teleosts are not entirely clear. Commonly accepted, they opsonize pathogens to enhance phagocytic clearance. Accordingly, rainbow trout (*Salmo gairdneri*) immunized with formalin-inactivated *Vibrio anguillarum* emulsified in Freund's complete adjuvant (FCA) resisted intraperitoneal administration of living *Vibrio anguillarum* several days after immunization. Thereby, a significant increase of CRP on phagocytic activity and opsonization demonstrated macrophage activation in the early stage of infection (19). Likewise, the expression of a CRP/SAP-like protein from ayu, *Plecoglossus altivelis* (PaCRP/SAP) was significantly upregulated following *Vibrio anguillarum* infection. *In vitro*, both Gram-negative and Gram-positive bacteria were agglutinated by PaCRP/SAP in a calcium-dependent manner. So far so good. Unexpectedly, however, the agglutination inhibited deposition of ayu complement 3 (PaC3) on the bacteria further inhibiting complement-mediated opsonization and phagocytosis by ayu monocytes/macrophages (20).

Recently, unexpected *in vivo* and *in vitro* anti-viral functions of the seven CRP (crp1-7) genes of zebrafish (*Danio rerio*) led to the discovery of a crp1-7/CRP1-7 primitive anti-viral functional diversity against spring viremia carp virus (SVCV) (21). Even before, it was demonstrated that CRP and complement behaved as acute phase proteins if stimulated by Cyprinid herpesvirus 3 (CyHV-3) infection, with an organ- and time-dependent response (22). The latter is a severe disease of common carp *Cyprinus carpio* and its ornamental koi varieties.

It came as little surprise, that parasites also have an impact on the expression of CRP. In the goldfish infected with *Trypanosoma carassii*, CRP and SAA exhibited the highest expression among several other acute phase proteins in liver, spleen and kidney. Recombinant goldfish CRP (rgfCRP) promoted complement-mediated lysis of trypanosomes *in vitro* further enhanced by addition of immune serum. However, neither the production of reactive oxygen nor nitrogen species by monocytes and macrophages, respectively, was affected (23). Both rat and human CRPs bound to sporozoites with subsequent inhibition of their *in vitro* development in hepatocytes. Specifically, the penetration of the sporozoite into the hepatocyte was prevented and parasite division suppressed by an antibody-like effect (31).

Already in the sixties, it was proposed that mouse CRP is an opsonin contributing to innate immunity to infection with *S. aureus* (32). 30 years later, the study by Szalai et al. provided evidence that CRP indeed contributed significantly to host defense: CRP transgenic mice experimentally exposed to *Streptococcus pneumoniae* had a significant better outcome compared to their nontransgenic littermates due to a substantial reduction of bacterial load. Furthermore, due to an increased CRP expression mediated by testosterone, male transgenics lived longer than females (33). *Vice versa*, CRP-deficient mice had a worse outcome following *Streptococcus pneumoniae* infection and could be protected either by reconstitution with isolated pure human CRP, or by anti-pneumococcal antibodies (39). Efforts to shed light on the mechanisms revealed that one of the hallmarks of CRP function, activation of the classical complement pathway, was not involved in protecting mice from infection (40).

Interestingly, in rats, treatment of platelets with CRP bestowed significant protection capacity against schistosomiasis in transfer experiments and obviously participated in the natural resistance of this species to schistosomal infection (41).

## Atherosclerosis

At a first glance, discussing CRP and atherosclerosis in conjunction with phylogeny and “lower” organisms seems to be absurd. On closer examination, however, the situation is somewhat different for the following reasons: (1) Establishing “lower” animal models of atherosclerosis is of great benefit, in particular with regard to large scale screening of potential therapeutic targets. For example, thanks to their unique properties (fertility, rapid development *ex utero*, transparency, lipid metabolism) zebrafish (*Danio rerio*) has become a promising animal model for vascular biology as well as screening and evaluation of drug therapy (49). Even before, it was stressed that *Caenorhabditis elegans, Drosophila melanogaster*, and *Danio rerio* are useful candidates for the identification of new pharmaceutical targets for metabolic diseases (50). (2) To unravel the diverse biological functions obviously associated with atherogenesis, a lot of transgenic mice and rabbit models had been used for translational research. The undoubted evidence obtained from these animal models was that plasma CRP levels were indeed elevated on the one hand and CRP was present already in initial atherosclerotic lesions on the other hand. However, the evidence concerning the net effect of CRP on initiation and progression of atherosclerosis is still lacking (44) leading over to (3) with excessive tissue-stranded modified lipoprotein during atherogenesis being a prime example of the misguided nutritional culture of the most highly evolved primate *Homo sapiens* with subsequent either beneficial or harmful (still to be clarified) participation of innate immunity with CRP as an important component (see below).

## Mechanistic Insights of CRP Function

Admittedly, the functions of CRP during inflammation are still a matter of debate; however, it was suggested that different conformations (native and non-native) of CRP have to be considered to unravel its functions. In particular, ligand recognition of CRP was supposed to be dependent on its conformation shifting at sites of inflammation (3). Other properties of CRP also depended on dissociation of its native pentameric conformation into the monomeric form (mCRP) (34, 51). As for the latter, the cholesterol binding sequence (CBS; a. a. 35-47) mediated the binding of mCRP to apolipoprotein B, complement component C1q, fibronectin, collagen, fibrinogen and, of course, cholesterol. Moreover, activation of endothelial cells by mCRP *in vitro* and induction of IL-6 *in vivo* was significantly reduced by CBS. The single sequence motif CBS obviously was a major recognition site of mCRP and a promising candidate for the regulation of mCRP effects (35).

Another exemplary development of a mechanistic insight of CRP function was given by the protection of mice by CRP from *Streptococcus pneumoniae* infection by the group of Agrawal: Initially, it was concluded that the CRP-mediated amelioration of bacterial load and the resulting protection was not to be connected in any way to CRP binding to the pathogen and subsequent complement activation. Including the notion that Fcγ receptors were not involved either (36), possible effects of CRP on cell-mediated cytotoxicity were favored (37). Shortly after, it was shown that administration of pneumococci must be followed by CRP not later than within a few hours. Otherwise, the protective effect of CRP was abolished suggesting a prophylactic rather than therapeutic effect of CRP (38). Subsequently, the phosphocholine (PCh)-binding pocket on CRP turned out to be decisive for the beneficial CRP effect during early pneumococcal infection of mice (45). This assumption was once more modified by the later statement that mice were protected against pneumococcal infection by both PCh-dependent and PCh-independent CRP effects (42).

## What Does it Mean?

Setting out for about 500 million years of evolution the most archaic physiological roles of CRP seemed to be detoxication of heavy metals and other chemicals followed or accompanied by an acute phase response and host defense against bacterial, viral as well as parasitic infection. So far, this seems to be conclusive. As for heavy metals, however, it is legitimate to ask whether this is really a physiological role and whether the amounts of heavy metals used *in vitro* are achievable in the body under *in vivo* conditions.

Concerning human diseases, the role of CRP was even more complex and ambiguous: On the one hand, in autoimmune conditions like Systemic Lupus Erythematosus (SLE), increased CRP levels contributed to efficient clearance of potential autoantigens (43). Monocyte subpopulations of severly injured trauma patients produced anti-inflammatory cytokines in response to acute phase concentrations of CRP (30). On the other hand, dead cell bound CRP is an important target for anti-CRP antibodies in patients with SLE increasing the production of cytokines by macrophages thus shifting the clearance process toward inflammation (52). In addition, CRP played a decisive role in secondary tissue damage in cardiac infarction (53) and, *vice versa*, apheresis of CRP could reduce damaged infarction area (54) questioning the black-and-white perception of CRP as a being invariably beneficial. Moreover, during phylogeny, unusual antigens have emerged coming along either as autoantigens like modified LDL (44, 55) or as foreign antigens like SARS-CoV-2 (56, 57). As already mentioned above, excessive tissue-stranded modified lipoproteins during atherogenesis is a prime example of an evolutionary emerging autoantigen due to misdirected food intake with subsequent interaction of CRP with modified LDL (55). However, the hitherto existing data are ambiguous making it impossible to draw a conclusion on potential beneficial or harmful effects of such interaction. Both the structural diversity of CRP and/or modified LDL might be responsible for the current ambiguity. For example, the mCRP present in atherosclerotic lesions may be the result of CRP binding to different ligands (58). The main implications of such ligand binding relate to foam cell formation on the one hand and complement activation on the other hand. As for the former, it was suggested that CRP inhibited foam cell formation by eLDL (enzymatically modified LDL) (59). Moreover, this inhibitory effect was boosted by phosphoethanolamine, which potentiated the binding of CRP to eLDL (60). As for the latter, the eLDL hypothesis contended modification of LDL by ubiquitous hydrolytic enzymes resulting in either atherosclerotic lesion initiation with reversion or progression according as there is a balance between cholesterol insudation and depletion or not. With regard to eLDL triggered complement activation, the subsequent effects of eLDL were ambivalent. The first CRP-dependent activation step dominated during early atherogenesis (lesion initiation with reversion by virtue of the capacity of CRP to bind factor H prohibiting the complement sequence at the stage of C3b/C5 thus sparing the deleterious terminal complement cascade), and the second CRP-independent activation step getting out of hand as eLDL accumulated over a critical threshold (lesion initiation with progression by completion of the terminal complement cascade). Of course, the effects of CRP on both foam cell formation and complement activation may considerably influence atherosclerotic lesion formation [reviewed in Torzewski (55, 61)].

**Figure 1:**
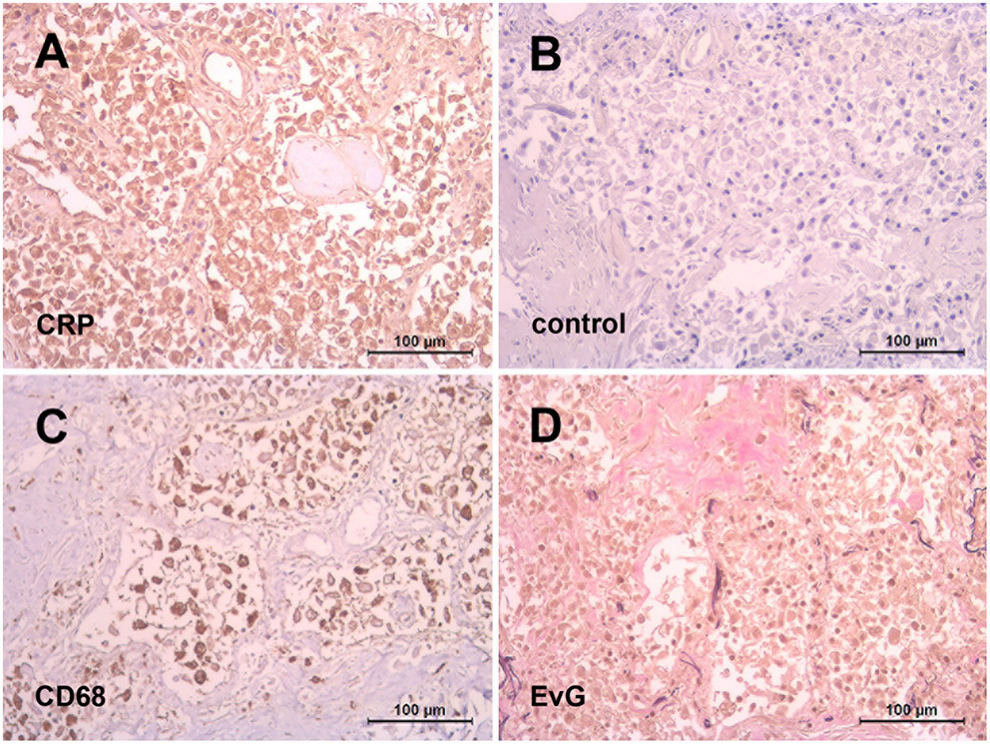
Abundant CRP expression in the lung after SARS-CoV-2 infection. Representative immunohistochemistry of paraffin embedded lung tissue from a patient died of COVID-19. Diffuse alveolar damage with intensive positive CRP staining (monoclonal antibody (mAb) clone CRP-8, Sigma) of macrophages as identified with mAb against CD68 (clone PG-M1, Dako) **(A,C)**. Negative control with an irrelevant isotype-matched mAb (FLEX, Dako) **(B)**. Interstitial pulmonary fibrosis illustrated by Elastica-van Gieson stain **(D)**.

Besides is importance as a prognostic factor of severity and mortality (62), an obviously harmful effect of CRP was unmask by the recent COVID-19 pandemic insofar as individual patients were treated successfully by selective CRP apheresis (56, 57). The rationale behind was that SARS-CoV-2 infection initiated an unhalted autoimmune response by CRP going along with macrophage and complement activation suspected to be responsible for pulmonary fibrosis and subsequent organ failure in COVID-19. This assumption might be illustrated by our preliminary observation of an abundant CRP expression in the lung of patients died of COVID-19 (Figure 1). The latter should be considered in particular with respect to the recently described immunological profiles of COVID-19 lungs (63) suggesting a complex interplay of innate and adaptive immunity underlying the clinical picture. It has to be emphasized, however, that other anti-inflammatory treatment options, for example blocking interleukin-6 or inhibiting the C3 and C5 activation also showed a promising preclinical effect (64).

## Conclusion

Coming back to the opening question whether CRP is functional in all humans and whether it is beneficial or harmful, about 500 million years of evolution teached us that there was a continuous interplay between emerging antigens and components of innate immunity. The examples of both atherogenesis and COVID-19 impressingly demonstrated that a component of ancient immunity like CRP should not be considered under identical “beneficial” auspices throughout phylogeny but might effect quite the reverse as well.

## Author Contributions

The author confirms being the sole contributor of this work and has approved it for publication.

## Funding

Sources of funding received are the Robert Bosch Stiftung, Stuttgart grant number P7. The APC was funded by Robert Bosch Gesellschaft für medizinische Forschung mbH.

## Conflict of Interest

The author declares that the research was conducted in the absence of any commercial or financial relationships that could be construed as a potential conflict of interest.

## Publisher's Note

All claims expressed in this article are solely those of the authors and do not necessarily represent those of their affiliated organizations, or those of the publisher, the editors and the reviewers. Any product that may be evaluated in this article, or claim that may be made by its manufacturer, is not guaranteed or endorsed by the publisher.
